# 2619. Impact of Positive Real-Time Polymerase Chain Reaction Respiratory Viral Panel on the Duration of Antimicrobials

**DOI:** 10.1093/ofid/ofad500.2232

**Published:** 2023-11-27

**Authors:** Akshith Dass, Yahira Diaz-Cardona, Sunita Patel

**Affiliations:** Cleveland Clinic Mercy Hospital, Canton, Ohio; Cleveland Clinic Mercy Hospital, Canton, Ohio; Cleveland Clinic Mercy Hospital, Canton, Ohio

## Abstract

**Background:**

Viral pathogens are increasingly identified as the most common cause of respiratory infections. Viral respiratory panels, such as the real-time polymerase chain reaction (RT PCR), have high specificity and sensitivity for infections due to detected pathogens and are a safe tool to detect viral pathogens. The American Thoracic Society (ATS) recognizes that detecting viral pathogens or mixed viral and bacterial infections in patients with lower respiratory infections can promote appropriate antibiotic use. Optimizing the use of antibiotics is critical to effectively treat infections, protect patients from harm caused by unnecessary antibiotic use, and combat antibiotic resistance. The primary objective of this study is to determine the impact of viral respiratory panel results on the duration of antibiotic use and outcomes in patients with respiratory tract infections.

**Methods:**

A retrospective cohort study of adult patients admitted to Cleveland Clinic Mercy Hospital from November 2021 through May 2022 with a diagnosis of suspected respiratory infection and ordered an RT PCR panel was performed. Patients with a confirmed bacterial co-infection were excluded. The primary outcome of this study is the median duration of antibiotic use. Secondary outcomes include all-cause hospital mortality, hospital length of stay, all-cause hospital readmission within 30 days, and *Clostridioides difficile* infection within 60 days.

**Results:**

A total of 113 patients were included in the study, of those, 44 patients had a negative viral respiratory panel and 69 patients had a positive viral respiratory panel. The patient population was predominantly male (53%), with a median age of 69 years. The RT PCR negative group had a higher proportion of patients with COPD and CHF compared to the RT PCR positive group (84% vs 38%, p< 0.001 and 50% vs. 28%, p=0.018, respectively). The primary outcome of median duration of antibiotics was significantly lower in the RT PCR positive group (0 [0-5] days vs. 6 [0-8] days, p=0.0001). There were no statistically significant differences observed between groups for any of the secondary outcomes.

**Figure:**
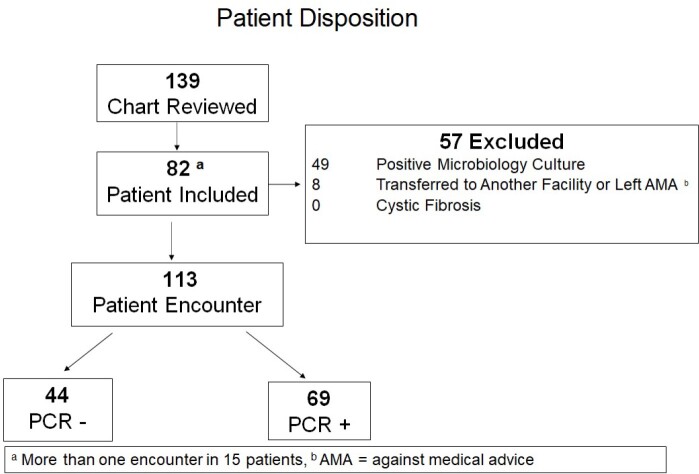

**Figure:**
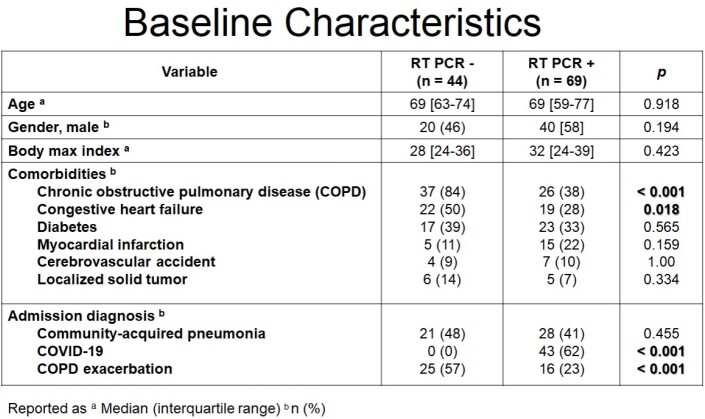

**Figure:**
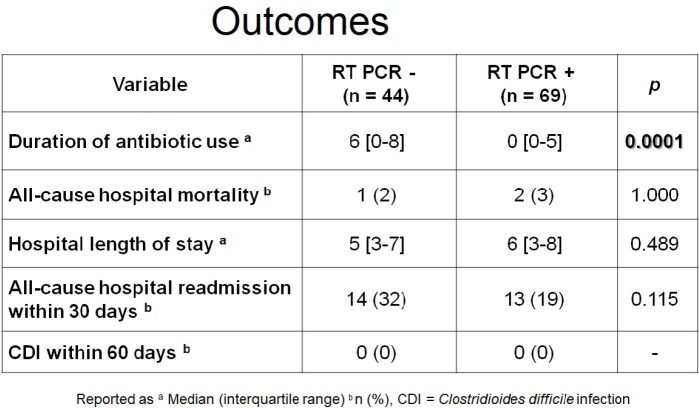

**Conclusion:**

A viral respiratory panel is a safe tool for de-escalation of inappropriate antibiotic use in patients with respiratory tract infections.

**Disclosures:**

**All Authors**: No reported disclosures

